# Optimal Thromboprophylaxis in Laparoscopic Bariatric Surgery in a Private Third-Level Center in Mexico City

**DOI:** 10.7759/cureus.86593

**Published:** 2025-06-23

**Authors:** David Lomeli-Reyes, Alejandro Martinez-Esteban, Natalia M Barron-Cervantes, Sofia Peña, Gonzalo Torres Villalobos, Alejandro D G. Gidi

**Affiliations:** 1 General and Gastrointestinal Surgery Service, Medica Sur Hospital, Mexico City, MEX; 2 Internal Medicine, Fundacion Clinica Medica Sur, Mexico City, MEX; 3 Bariatric Surgery Clinic, Medica Sur Hospital, México City, MEX; 4 General and Gastrointestinal Surgery, Angeles Health System, Mexico City, MEX

**Keywords:** bariatric surgery, deep vein thrombosis, enoxaparin, heparin, laparoscopic approach, pulmonary embolism, thromboprophylaxis, venous thromboembolism

## Abstract

Venous thromboembolism (VTE), which includes deep vein thrombosis (DVT) and pulmonary embolism (PE), is a significant source of morbidity and mortality in patients undergoing bariatric surgery. As the prevalence of obesity and metabolic syndrome continues to rise, particularly in Mexico, the number of bariatric procedures has also increased. However, optimal strategies for thromboprophylaxis in laparoscopic bariatric surgery remain unclear, with limited data specific to the Mexican population. This study aimed to evaluate the incidence of VTE as the primary outcome and the safety and associated complications of different thromboprophylaxis strategies in patients undergoing laparoscopic bariatric surgery at a tertiary care center in Mexico City. Specifically, the study compared mechanical prophylaxis to combined mechanical and pharmacological prophylaxis. The findings provide objective data on the effectiveness of these approaches in preventing VTE and related complications in this patient population.

## Introduction

Obesity represents a global metabolic and nutritional challenge, imposing a significant strain on healthcare resources [1]. Over recent decades, there has been a dramatic increase in the incidence of obesity, prompting the exploration of bariatric interventions, which have demonstrated efficacy in reducing body weight, ameliorating comorbidities, and enhancing the quality of life for affected individuals [2]. In Mexico in 2024, 70% of the population presented with overweight or obesity [3]. Notably, it is important to mention that obesity stands as an independent risk factor for venous thromboembolism (VTE), with a body mass index (BMI) ≥30 kg/m2 correlating with a twofold increase in VTE risk [4]. Patients undergoing bariatric surgery are particularly predisposed to VTE, although with a relatively low overall incidence; thromboembolic events persist as a primary cause of postoperative mortality in the realm of bariatric surgical procedures [5-7]. 

Despite the rising burden of obesity-related diseases in Latin America, data on VTE prevention strategies in bariatric surgery remain scarce in the Hispanic literature, particularly in the Mexican context. This lack of information is especially relevant given that healthcare systems, patient demographics, and clinical practices can differ substantially from those in high-income countries where most of the available data originate. For instance, Mexican patients may present with different comorbidity profiles, such as higher rates of uncontrolled diabetes or delayed access to specialist care. Additionally, resource availability, institutional protocols, and adherence to postoperative follow-up may vary across public and private healthcare settings in Mexico, potentially influencing the risk-benefit balance of pharmacologic and mechanical thromboprophylaxis strategies.

To support individualized VTE prevention in surgical patients, risk stratification tools such as the Caprini Risk Assessment Model are commonly used. Despite its widespread use, there is limited evidence evaluating the real-world impact of risk-based, tailored thromboprophylaxis regimens-particularly in Latin American bariatric surgery populations. In response to this gap, we conducted a retrospective cohort study of patients who underwent laparoscopic bariatric surgery between March 2013 and September 2020 at Fundación Clínica Médica Sur. Thromboprophylaxis was administered to all patients before surgery and maintained through hospital discharge; continuation after discharge was determined based on individual risk profiles. Patients were stratified using the Caprini score, and those with moderate or high risk received combined pharmacological (weight-adjusted low molecular weight heparin) and mechanical prophylaxis, while low-risk patients received mechanical prophylaxis alone.

The primary aim of this study was to assess the incidence of postoperative VTE within 30 days of surgery. Secondary objectives included evaluating bleeding events and other safety outcomes associated with each prophylaxis strategy. By comparing mechanical prophylaxis alone versus combined mechanical and pharmacological regimens, we aimed to explore whether tailored thromboprophylaxis based on preoperative risk stratification could reduce VTE incidence without increasing adverse events. Additionally, this study contributes regional data to the limited literature on VTE prevention in bariatric surgery and allows comparison with international clinical standards.

## Materials and methods

A retrospective study was carried out where patients who underwent laparoscopic bariatric surgery at the Fundación Clínica Médica Sur, a third-level hospital, were studied from March 2013 to September 2020. The inclusion criteria of this study were: all adult patients (≥18 years) with body mass index (BMI) ≥ 35 kg/m2 with associated comorbidities and patients with BMI ≥ 40 kg/m2 without comorbidities who underwent bariatric surgeries. Within this group, all patients who underwent a gastric bypass, gastric band, or gastric sleeve through a laparoscopic approach were included. The following exclusion criteria were used: incomplete clinical records, patients < 18 years of age, patients with a BMI < 35 kg/m2, and patients with a BMI ≥ 35 kg/m2. without comorbidities. 

Objectives

The primary objective of this study was to determine the incidence of venous thromboembolism (VTE) in patients undergoing laparoscopic bariatric surgery. The secondary objectives were to evaluate the safety and rate of complications associated with different thromboprophylaxis strategies, specifically comparing mechanical prophylaxis alone versus combined mechanical and pharmacological prophylaxis. We aimed to assess whether the addition of pharmacological prophylaxis was associated with a reduction in VTE incidence and to examine any differences in bleeding or other adverse events between the groups. This study was designed as a descriptive, exploratory analysis; no formal hypothesis testing was conducted due to the low number of VTE events. However, we considered a clinically meaningful difference to be a reduction in VTE incidence without a disproportionate increase in bleeding-related complications.

Hypothesis

Given that patients undergoing laparoscopic bariatric surgery commonly present with comorbidities such as obesity, hypertension, diabetes, and metabolic syndrome-which are known risk factors for venous thromboembolism (VTE)-we hypothesize that the use of combined mechanical and pharmacological thromboprophylaxis will be associated with a lower incidence of postoperative VTE compared to mechanical prophylaxis alone. Furthermore, we anticipate that adherence to thromboprophylaxis protocols will not lead to a significant increase in bleeding or other complications, thereby supporting the safety and effectiveness of a combined prophylactic approach in this high-risk surgical population.

Study design

For this study, patients undergoing laparoscopic bariatric surgery were prescribed thromboprophylaxis in the preoperative and immediate postoperative periods until discharge from the hospital. Patients in this cohort were divided into two groups; one group was only treated with mechanical thromboprophylaxis, which included compression socks and pneumatic compression stockings. The second group was treated with mechanical thromboprophylaxis and with a BMI-adjusted dose of low-molecular-weight heparin (LMWH) at 40-60 mg once daily (10:00 a.m.) subcutaneously was administered for two to three days, accompanied by mechanical thromboprophylaxis initiated in the preoperative period. This regimen was based on institutional protocol. 

The decision to use either mechanical prophylaxis alone or to combine it with pharmacological prophylaxis was based on each patient's thromboembolic risk, which was evaluated using the Caprini score. This scoring system is specifically designed to assess the risk of VTE, particularly in surgical patients. It is mainly applied to preoperative patients, especially those undergoing non-orthopedic surgeries, making it particularly relevant for assessing perioperative risk in this cohort [8-10]. According to this risk scoring system, patients are stratified as follows: scores of 0 to one indicate low risk, two indicate moderate risk, three to four indicate high risk, and scores of five or higher represent very high risk. Only patients with a score of two or higher (i.e., moderate, high, or very high risk) received pharmacological prophylaxis in addition to mechanical methods. Patients classified as low risk (score 0 to one) received mechanical prophylaxis alone. To establish the comorbidities, patients needed to present a diagnosis signed by an accredited medical doctor or by one of the surgeons involved in this study. 

Adherence to both mechanical and pharmacologic thromboprophylaxis was carefully monitored throughout the hospital stay and after discharge. During hospitalization, compliance with pharmacologic prophylaxis was verified through nursing records documenting the administration of prescribed medications, while adherence to mechanical methods was observed and recorded by the clinical staff. Adherence to the post-discharge prophylaxis regimen was assessed during follow-up at seven and 30 days via structured telephone interviews conducted by trained staff using a standardized script. The interviews included closed-ended questions regarding medication adherence, side effects, and symptom onset suggestive of VTE or bleeding events.

VTE events were identified clinically and confirmed through imaging: symptomatic deep vein thrombosis (DVT) was evaluated using duplex ultrasonography, and pulmonary embolism (PE) was confirmed with contrast-enhanced computed tomography pulmonary angiography (CTPA). Institutional diagnostic criteria adhered to standard international guidelines. Bleeding complications were defined according to the International Society on Thrombosis and Haemostasis (ISTH) criteria, which include bleeding requiring clinical intervention, transfusion, or extended hospitalization. Minor self-limited bleeding or asymptomatic drops in hemoglobin were not classified as bleeding complications. Asymptomatic cases were not routinely screened with image studies and, therefore, were not included in the analysis. 

For statistical analysis, the SPSS Statistics software version 26.0.1 (2018, IBM Inc., Armonk, NY) was employed. Categorical variables were delineated in terms of frequencies and proportions, while continuous variables were expressed as medians and interquartile ranges (IQR). Categorical variables were compared using Fisher’s exact test, and continuous variables using Student’s t-test or Mann-Whitney U test, as appropriate. Statistical significance was defined as p < 0.05. Given the low number of events, we did not conduct a formal sample size or power calculation. Missing data were handled using complete-case analysis.

## Results

**Figure 1:**
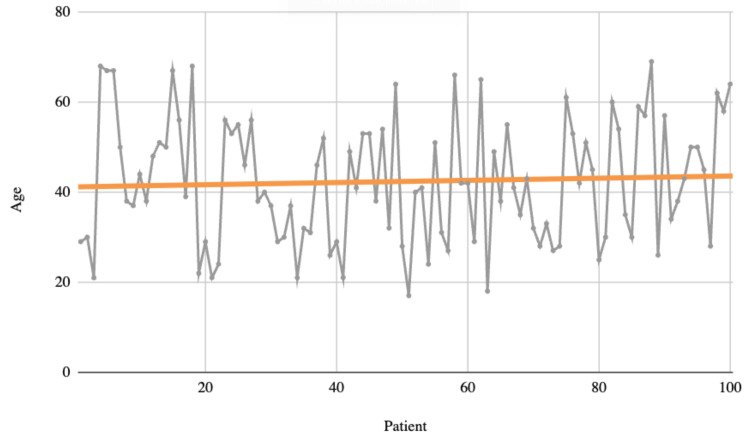
Age of the patients. Line graph depicting the age (Y axis) against the patients (X axis, N = 100). Horizontal orange line representing the median age of the patients, which was 42 years (SD ± 10 years).

This study cohort included 100 patients with a mean age of 42 years (Figure 1), with a predominance of females (64%, n=64). The cohort was divided into two groups: patients who received mechanical prophylaxis only (48%, n=48) and those who received both mechanical prophylaxis and pharmacoprophylaxis (52%, n=52) (Table 1). This classification was based on the preoperative thromboembolic risk assessment, determined by the Caprini score as outlined in the methodology. All patients were followed up immediately and up to 30 days postoperatively. Additionally, all patients received mechanical prophylaxis and initiated early deambulation on the day of surgery.

**Table 1:** Demographic data and clinical characteristics. Table presenting the patients’ characteristics in the first column, including age, sex, weight, type of surgery presented, surgical approach and comorbidities. The second column shows the number of patients that presented with each characteristic and the percentage of presentation considering 100 as the total number of subjects studied. LMWH = low molecular weight heparin.

Characteristics	Total patients (N = 100)	Percentage of presentation
Female sex	64	64%
Male sex	36	36%
Type of laparoscopic bariatric surgical procedure		
Gastric sleeve	31	31%
Gastric band	4	4%
Roux-en-Y gastric bypass	66	66%
Comorbidities presented in the studied population		
Type 2 diabetes	45	45%
Systemic arterial hypertension	35	35%
Hypothyroidism	14	14%
Asthma	7	7%
Obstructive sleep apnea	13	13%
Dyslipidemia	29	29%
Chronic obstructive pulmonary disease	6	6%
Chronic deep vein thrombosis	5	5%
Metabolic syndrome	45	45%
Thromboprophylaxis utilized		
Only mechanical	48	48%
Mechanical + LMWH	52	52%

**Figure 2:**
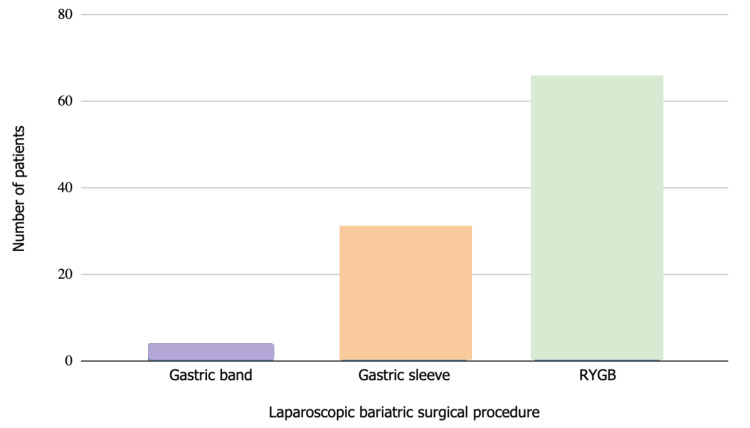
Type of laparoscopic bariatric surgical procedure. Bar graph representing the number of patients that underwent each surgical procedure (Y axis) against the type of laparoscopic bariatric surgical procedure (X axis). Roux-en-Y gastric bypass (RYGB) was the most common procedure performed (66 patients, 66%), then gastric sleeve (31 patients, 31%) and finally, the least performed procedure was gastric banding (4 patients, 4%). N = 100.

The majority of patients (66%, n=66) underwent laparoscopic Roux-en-Y gastric bypass (RYGB), while patients undergoing gastric banding (4%, n=4) were the minority of the study population. The rest of the population underwent a gastric sleeve (31%, n=31) (Figure 2).

**Figure 3:**
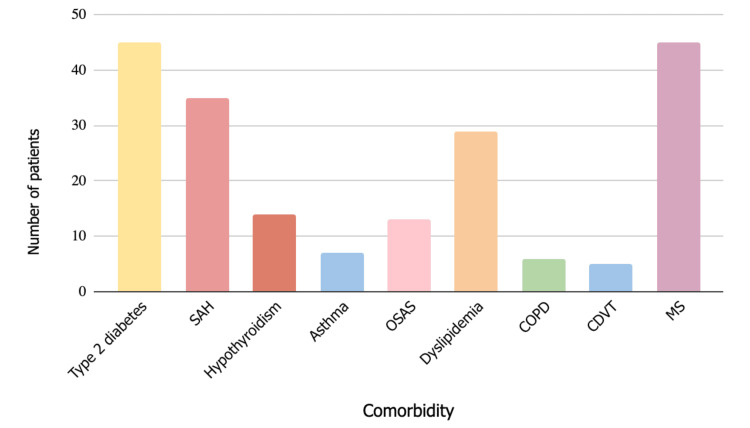
Comorbidities presented in the studied population. Bar graph representing the number of patients that have been diagnosed with each comorbidity (Y axis) against the type of comorbidity presented (X axis). The most common comorbidities presented in the population were type 2 diabetes (45 patients, 45%) and metabolic syndrome (MS) (45 patients, 45%). The remaining comorbidities, arranged in descending order of prevalence, include the following: systemic arterial hypertension (SAH) (35 patients, 35%), dyslipidemia (29 patients, 29%), hypothyroidism (14 patients, 14%), obstructive sleep apnea syndrome (OSAS) (13 patients, 35%) , asthma (7 patients, 7%), chronic obstructive pulmonary disease (COPD) (6 patients, 6%) and chronic deep vein thrombosis (CDVT) (5 patients, 5%). N = 100.

Median body weight was 110 kilograms (IQR 80-150.1). The BMI of 68 patients (68%) was 35-39 kg/m2, and 32 patients (32%) were > 40 kg/m2. Comorbidities presented and reported in this study were type 2 diabetes, systemic arterial hypertension (SAH), hypothyroidism, asthma, obstructive sleep apnea syndrome (OSAS), dyslipidemia, chronic obstructive pulmonary disease (COPD), chronic deep vein thrombosis (CDVT), and metabolic syndrome. The most common comorbidities presented were type 2 diabetes (45%, n=45) and metabolic syndrome (MS) (45%, n=45), and the least presented comorbidity was chronic deep vein thrombosis (5%, n=5) (Figure 3).

**Figure 4:**
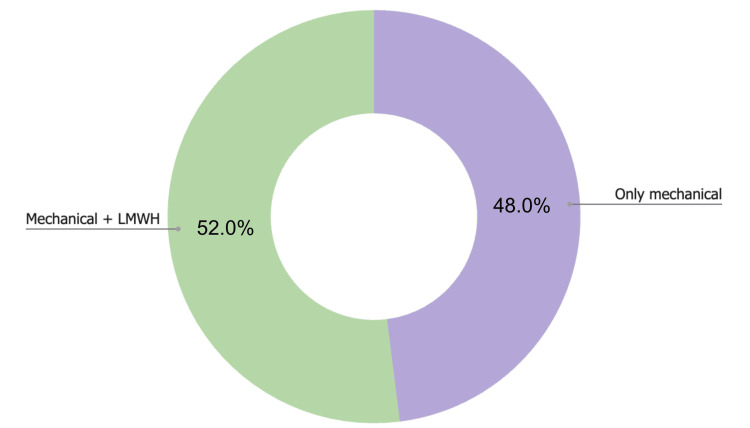
Thromboprophylaxis regimen. Donut chart representing the types of thromboprophylaxis regimens utilized. The first group (green) represents the percentage of patients that were under a mechanical and pharmacological regimen with low molecular weight heparin (LMWH) (52 patients, 52%). The second group (purple) represents the percentage of patients that were only under a mechanical thromboprophylaxis (48 patients, 48%). N = 100.

As mentioned in the methodology, patients were divided into two main subgroups based on their preoperative Caprini scores. Patients with a low preoperative risk received only mechanical prophylaxis and early deambulation (48%, n=48), while those with moderate or high risk were given early deambulation, mechanical prophylaxis and a BMI-adjusted dose of LMWH 40-60 mg once daily subcutaneously for a range of two to three days (52%, n=52) (Figure 4).

Of all the patients studied, only two cases of deep venous thrombosis occurred (2%, n=2); these cases were diagnosed through venous duplex ultrasound. No cases of pulmonary embolism were found. These cases occurred in women with a BMI >40 kg/m2, both of whom underwent RYGB and were only on mechanical thromboprophylaxis. It is also important to note that no bleeding complications or adverse effects related to LMWH were observed.

## Discussion

Bariatric surgery has become a key therapeutic approach for managing severe obesity, a condition that has reached epidemic proportions worldwide. Obesity, now recognized as a multifactorial chronic disease, is closely linked to metabolic syndrome and increases the risk of various life-threatening conditions such as cardiovascular disease, diabetes, and certain cancers. As the prevalence of obesity continues to rise, so does the demand for effective interventions like bariatric surgery, which has proven to be one of the most effective treatments for significant and sustained weight loss, leading to improvements in metabolic health and overall quality of life. However, like any major surgical procedure, bariatric surgery carries the risk of postoperative complications, including thrombotic events [11]. Obese patients are particularly vulnerable to these complications due to their increased baseline risk for thrombosis. Preventive measures, such as appropriate thromboprophylaxis, are essential in reducing this risk and ensuring better surgical outcomes. Gastric sleeve (GS) currently ranks as the most common bariatric procedure performed globally, further highlighting the growing importance of bariatric surgery in addressing severe obesity and its associated health risks [12]. 

Patients undergoing bariatric surgery have a higher risk of VTE since the main factors include obesity and surgery [13-16]. Recommendations in the immediate postoperative period include early postoperative deambulation, elastic compression devices, and the use of pneumatic compression devices and the use of thromboprophylaxis [17]. The omission and lack of compliance with antithrombotic measures according to their specific indications lead to an increased risk of complications in the immediate postoperative period [18]. The immediate postoperative period covers the first 72 hours after surgery. The incidence of VTE, presenting as DVT or PE, after bariatric surgery has been reported between 0.5 and 2% [19], increasing in-hospital morbidity and mortality. Most series evaluating prophylactic strategies for bariatric patients include some form of mechanical prophylaxis. Due to concerns of bleeding complications associated with chemoprophylaxis (2% incidence of bleeding complications in a recent systematic review when a standardized definition of bleeding was used) [2], several studies have examined the use of mechanical compression only in bariatric surgery patients. 

A retrospective study of 1692 patients evaluated VTE rates comparing low molecular weight heparin (LMWH) 40 mg twice daily and sequential compression devices (SCD) (N 1⁄4,435) with patients receiving SCD and early deambulation (within 2 hours of arrival at the room) only (N 1⁄4 1257). This study represented a change in the authors' practice protocol over time and was not a randomized trial. These authors reported rates of DVT and PE of 1.6% and 1.1%, respectively, in patients receiving LMWH and SCD compared with a rate of DVT and no PE of 0.4% in patients receiving mechanical prophylaxis and early deambulation. Bleeding complications were higher in the LMWH group (4.8%) compared to the mechanical prophylaxis group (0.4%) [20]. The generalizability of these results is limited because it is a single practice experience with fewer complications over time and higher mean BMI, and longer surgical times in the group that received chemoprophylaxis. Another study reported a retrospective analysis of 957 consecutive patients undergoing laparoscopic Y-Roux gastric bypass surgery who did not receive pharmacological treatment for VTE prevention [15]. Calf SCDs were placed before surgery, and early, frequent deambulation was encouraged. The authors reported 30-day DVT and PE rates of 0.31% and 0.10%, respectively, and a bleeding complication rate of 0.73%. The two previous studies excluded patients at high risk of VTE. The authors suggest that mechanical prophylaxis is sufficient for patients without a strong personal or family history of VTE events or a known hypercoagulable state. It should also be noted that the reported VTE rates were based on symptomatic patients who underwent diagnostic testing and did not undergo routine imaging or screening.

In 2007, the American Society for Metabolic and Bariatric Surgery (ASMBS) initially recommended a multifaceted approach to thromboprophylaxis encompassing early deambulation, the utilization of intermittent compression devices, and, unless contraindicated, the adoption of pharmacological thromboprophylaxis [21]. The reported incidence of symptomatic DVT and PE in bariatric patients varies from 0% to 5.4% and 0% to 6.4%, respectively, with larger and more extensive studies indicating rates <1%, aligning with those observed in other elective surgical contexts [22]. Despite this, there remains a dearth of accurate, evidence-based risk assessment tools for VTE in the specific population of bariatric patients, necessitating consideration of various risk factors when formulating prophylactic strategies. The Longitudinal Assessment of Bariatric Surgery (LABS) study in 2009 documented a 30-day incidence of VTE complications at 0.4%, with a notable association between increased body weight and elevated risk. However, the study concluded that there existed insufficient evidence to advocate for a specific recommendation regarding VTE prophylaxis post-bariatric surgery, particularly concerning the adjunctive use of chemoprophylaxis alongside mechanical measures [15, 23]. Subsequent data gleaned from the longitudinal bariatric outcomes database, encompassing prospectively obtained information from 74,000 patients, revealed a VTE incidence of 0.42% within the initial 90 days post-surgery. Notably, over 70% of patients received chemoprophylaxis, with mechanical thromboprophylaxis administered to 93%, yet the majority of VTE events (73%) manifested after hospital discharge, with a significant proportion occurring within the first 30 days [16]. 

Furthermore, a review of 10 autopsies conducted post-bariatric surgery unveiled pulmonary embolism as the established direct cause of death in 30% of cases; however, it was elucidated that 80% of those instances were initially misdiagnosed as pulmonary edema [24]. Our research findings are in concordance with the scholarly works referenced earlier, thereby corroborating the relevance of these results and recommendations within the context of the Mexican population. Specifically, our study confirms that the prevalence of VTE at 2% among the Mexican population aligns closely with the corresponding percentages reported on a global scale. By acknowledging the consistent prevalence of VTE across different populations, healthcare practitioners and policymakers can enhance their efforts to devise comprehensive strategies aimed at mitigating the burden of this condition within the Mexican healthcare framework. 

These findings carry important implications for bariatric centers in Mexico and other middle-income countries, where resource constraints, patient profiles, and health system structures may differ from those in high-income settings. Our observed incidence of VTE, although low, supports the potential utility of individualized risk stratification tools such as the Caprini score to optimize thromboprophylaxis regimens. Tailoring prophylaxis according to patient-specific risk allows for balancing the benefits of VTE prevention against the risks of bleeding complications. Notably, our results suggest that mechanical prophylaxis combined with early mobilization may be an effective and feasible approach in selected patients, particularly in contexts where routine pharmacological prophylaxis may be limited by cost or safety concerns. Integrating such evidence-based, risk-adapted strategies into national clinical protocols could enhance patient safety while accommodating regional healthcare realities, ultimately aligning local practice with international standards.

It is important to highlight that both cases of deep vein thrombosis in our cohort occurred in female patients with a BMI greater than 40 kg/m² who received only mechanical thromboprophylaxis and underwent Roux-en-Y gastric bypass (RYGB). While these observations may raise concerns about the adequacy of prophylaxis in this subgroup, it is critical to note that both patients had low overall Caprini scores below the threshold generally recommended for pharmacologic thromboprophylaxis. The Caprini score emphasizes cumulative risk factors rather than isolated variables such as BMI alone. In these cases, despite elevated BMI, the absence or minimal presence of other risk factors resulted in a low total score, consistent with guideline-based decisions to withhold anticoagulation to minimize bleeding risk. This underscores the importance of comprehensive risk assessment rather than relying on single risk factors when determining thromboprophylaxis strategies.

This study presents several inherent limitations that must be considered when interpreting its findings. The retrospective design of this study inherently introduces potential limitations, including selection and information biases stemming from the reliance on non-standardized and potentially incomplete medical records. Variability in documentation practices may have affected the accuracy and consistency of data collection, particularly for complications or outcomes not systematically recorded. Additionally, no formal sample size justification or power calculation was performed before analysis, which limits the ability to draw robust conclusions, especially for low-incidence events such as VTE, which typically occur in approximately 2% of bariatric surgery patients. As a result, the 100-patient cohort may lack the statistical power needed to detect significant differences or associations related to rare outcomes. Future prospective studies with standardized data collection and appropriate sample size calculations are warranted to validate these findings and more accurately assess the effectiveness of thromboprophylaxis strategies in this population. Additionally, the single-center nature of the study, conducted in a private tertiary care hospital in Mexico City, restricts the generalizability of the findings to broader, more diverse populations, including those in public healthcare settings or rural areas. 

The study’s single-center setting at a private tertiary hospital in Mexico City also limits the generalizability of our findings. Patient demographics and healthcare delivery may differ significantly in public institutions or rural areas, underscoring the need for multicenter investigations that capture broader patient populations. Additionally, our methodology did not include routine postoperative screening for asymptomatic VTE, relying instead on imaging only in symptomatic patients. This approach may have resulted in an underestimation of VTE incidence due to missed subclinical events. Nonetheless, no clinical indications of undiagnosed VTE emerged during follow-up. Our 30-day postoperative monitoring period aligns with accepted standards for assessing surgical complications, capturing the majority of clinically significant VTE events, which typically manifest within this timeframe. The combination of in-hospital surveillance and structured post-discharge interviews enhanced the detection of symptomatic events. Still, we acknowledge the potential value of extended follow-up periods in future studies to identify late-onset thromboembolic complications, especially in higher-risk patients or those with reduced mobility after discharge.

Our strengths include the use of a standardized and validated risk stratification tool (the Caprini score) to tailor thromboprophylaxis, ensuring an evidence-based approach to patient care. We offer comprehensive baseline data on a bariatric surgery population from a Latin American tertiary care center, addressing a gap in regional literature. Additionally, our structured follow-up protocol, combining in-hospital surveillance with post-discharge interviews, enhances the accuracy of postoperative outcome assessment within the important 30-day window. These elements contribute valuable insights applicable to similar middle-income country settings. All of these strengths and limitations, now explicitly discussed, highlight the necessity of prospective randomized trials to more conclusively determine the comparative effectiveness of different prophylaxis strategies in bariatric surgery patients.

## Conclusions

Our study provides descriptive data consistent with international literature recognizing VTE as a significant postoperative complication in bariatric surgery and highlights the importance of standardized, evidence-based prevention protocols. The findings illustrate the use of preoperative risk stratification tools, such as the Caprini score, to guide individualized thromboprophylaxis in clinical practice. Within our cohort, both VTE cases occurred in high-BMI female patients who underwent RYGB and received only mechanical prophylaxis. Although this observation invites further inquiry into whether factors like BMI >40 kg/m² or specific surgical procedures such as RYGB should influence prophylaxis decisions beyond Caprini scoring, our study’s design and sample size do not allow for definitive conclusions or protocol recommendations. These results emphasize the need for further prospective research to explore these questions and assess the potential impact of tailored prophylactic strategies. Meanwhile, our data support the continued focus on comprehensive preoperative assessment and adherence to individualized thromboprophylaxis regimens aimed at minimizing VTE-related complications.
